# Development and Validation of a Micellar Capillary Electrophoresis Method for Determination of IFNβ-1b in Lyophilized Formulation of a Biosimilar Product

**Published:** 2015

**Authors:** Manuchehr Dadgarnejad, Hosein Rastegar, Hooshmand Ilka, Maryam Shekarchi, Nooshin Adib, Mahmood Alebouyeh, Nadia Keypour, Shahram Shoeibi, Farzad Kobarfard, Mohammad Reza Fazeli

**Affiliations:** a *Center of Food and Drug Control References Laboratories (CFDCRL), Food and Drug Organization (FDO), Ministry of Health and Medical Education (MOH), Tehran, Iran. *; b *Department of Drug and Food Control, Pharmaceutical Quality Assurance Research Center, Faculty of Pharmacy, Tehran University of Medical Sciences (TUMS), Tehran, Iran.. *; c *Research and Development Department, Zistdaru Danesh Co. Ltd., No. 1462, North Kargar Street, Tehran, Iran. *; d *Department of Medicinal Chemistry, School of Pharmacy, Phytochemistry Research Center, Shahid Beheshti University of Medical Sciences, Tehran, Iran.*

**Keywords:** Determination, Micellar electrokinetic chromatography (MEKC), Interferon, Ziferon®

## Abstract

Human interferons (IFNs) are key cytokines secreted by immune system. They have several effects such as antiviral and anti tumors activity, activating immune cells and healing of multiple sclerosis. The type IFNs present in humans are *α* *,β* and Υ. IFN *β* is a polypeptide, normally produced by fibroblasts and seems to be more species-specific than IFN. Structural properties of IFNs are important for their biologic effects. There are a few analytical techniques for separation, identification and determination of IFNs in its formulations such as mass spectroscopy, RP-HPLC and capillary electrophoresis (CE). In this study we used Micellar Electrokinetic Chromatography (MEKC) as a unique mode of CE because of its capability to separate neutral as well as charged solutes. We used sodium tetraborate (Borax) as buffer without any modifier and sodium dodecyl sulfate (SDS) as surfactant. The optimum MECK running buffer consisted of Borate 50 Mm; SDS 20 mM pH =9.6. The validated method was used for determination of the IFN β-1b formulation which is manufactured in Iran. From 9 collected different batches, all of them had acceptable potency as claimed on their label with average 102.25 ±10.030 %. This is the first time that a MEKC method is introduced for quantification of IFN β-1b in its pharmaceutical dosage forms. The method is reliable safe, rapid and accurate.

## Introduction

Nowadays, more than 100 pharmaceutical proteins have been approved and several hundred are currently in clinical trials. This development increases the demand for suitable methods of analysis that allow not only the separation and quantification of impurities and possible degradation products, but also their identification and, preferably, their physicochemical characterization (1). Furthermore, conformational integrity is also an important issue, and therefore analytical methods are needed that allow detection of proteins in their native, nondenatured state. Yet, another advantage of using on denaturing conditions is that artifacts caused by denaturation may be avoided.

**Figure 1:**
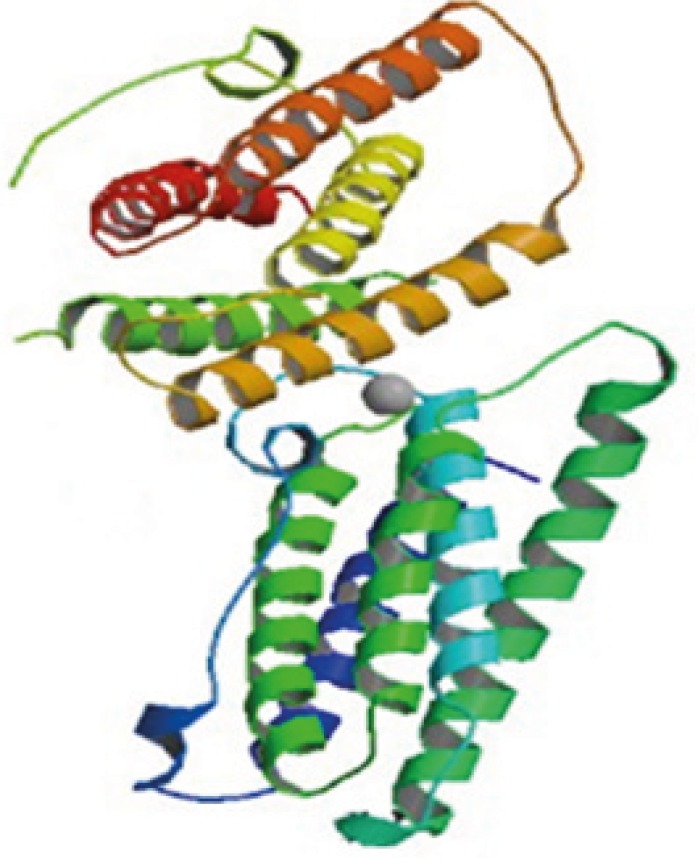
Human IFN ß-1b (165 residues), cysteine 17 is substituted with serine. Produced in E. coli, no carbohydrates, MW=18.5Kd C_908_H_1408_N_246_O_253_S_6_

IFN *ß*1b is a purified, sterile, lyophilized protein preparation produced by recombinant DNA techniques. IFN ß1b is manufactured by bacterial fermentation of a strain of Escherichia coli that bears a genetically engineered plasmid containing the gene for human IFN *ß*1b ser17. The native gene was obtained from human fibroblasts and altered in a way that substitutes serine for the cysteine residue found at position 17. IFN ß1b has 165 amino acids, isoelectric point of 9.2 and an approximate molecular weight of 18,500 Daltons. It does not include the carbohydrate side chains found in the natural material. The specific activity of IFN β-1b is approximately 32 million international units (IU)/mg of protein (1,2,3).

It is marketed as lyophilized vial and each vial contains 0.3 mg of IFN ß1b. The unit measurement is derived by comparing the antiviral activity of the product to the World Health Organization (WHO) reference standard of recombinant human IFN ß1b. Mannitol, USP and Albumin (Human), USP (15 mg each/vial) are added as stabilizers (4).

Capillary electrophoresis (CE) is a powerful separation tool, which in principle is very well suited for the analysis of proteins (5-8). Analyses in CE are relatively fast and separation efficiencies can be quite high (9,10). The separation mechanism is based on charge-to-size ratio of a compound, which makes it complementary to methods, such as reversed-phase liquid chromatography (RPLC) (11,12). Moreover, analyses in CE are normally performed using aqueous background electrolytes (BGEs), and, when required, the pH and temperature of separation can be chosen closely to physiological conditions. Separations in CE take place in an open tube in the absence of a stationary phase, which, in contrast to LC, further decreases the risk of protein denaturation due to interactions (13). Electrokinetic chromatography (EKC) is a family of electrophoresis techniques named after electrokinetic phenomena, which include electroosmosis, electrophoresis, and chromatography. Micellar electrokinetic chromatography (MEKC) is a mode of EKC in which surfactants (micelles) are added to the buffer solution. Surfactants are molecules which exhibit both hydrophobic and hydrophilic character. They have polar “head” groups that can be cationic, anionic, neutral, or zwitterionic and they have nonpolar, hydrocarbon tails. The formation of micelles or “micellization” is a direct consequence of the “hydrophobic effect.” The surfactant molecules can self-aggregate if the surfactant concentration exceeds a certain critical micelle concentration (CMC). The hydrocarbon tails will then be oriented toward the center of the aggregated molecules, whereas the polar head groups point outward. Micellar solutions may solubilize hydrophobic compounds which otherwise would be insoluble in water. The front cover picture shows an aggregated SDS molecule. In the center of the aggregate, p-fluoro toluene is situated depicting the partitioning of a neutral, hydrophobic solute into the micelle. Every surfactant has a characteristic CMC and aggregation number, i.e., the number of surfactant molecules making up a micelle (typically in the range of 50-100).The size of the micelles is in the range of 3 to 6 nm in diameter; therefore, Micellar solutions exhibit proper-ties of homogeneous solutions. Micellar solutions have been employed in a variety of separation and spectroscopic techniques. CE can also be coupled to various types of detection systems such as UV absorbance, laser-induced fluorescence (LIF) and mass spectrometry (MS).

 Several methods have been used for the analysis of content and purity of rhIFN (recombinant human IFN), e.g., reversed-phase, ion-exchange and size-exclusion LC (1,15,16). CE also shows great potential for the analysis of rhIFN and its degradation products (1,16). Recently, a CE-based method for detection of charge variants in rhIFN was incorporated in the European Pharmacopeia. Here we present an improved CE method for the characterization of IFN in pharmaceutical preparations.

## Experimental

*Reagents and materials*

IFN final container References was purchased from Chiron corporations (Emeryville, Ca, USA) and it was dissolved (300) in 1 mL water. Methanol (HPLC grade), Acetonitril (HPLC grade), 2-Propanol (HPLC grade) were from Merck (Darmstadt, Germany).All other chemicals were analytical grade and purchased from Merck (Darmstadt, Germany) throughout the study. The pH of the buffer solutions was adjusted by adding sodium hydroxide solution and was monitored using a pH meter Metrohm 798MPT Titrino.

*Apparatus and related procedures*

The separation of IFN was carried out using a capillary electrophoresis system (7100 Agilent, G7100A CE instrument Serial No .De 94300118) equipped with photodiode array detection (DAD) system. Instrumentation, system control and data analyses were carried out using a PC with the accompanying software (Chemstation Software, Revision B .04.02 Driver Service Pack 1). The electrophoretic separations were carried out in an uncoated fused-silica capillary (Ext .light Path CE CAP 50 µm ID, 56 cm Agilent Technologies) using a suitable running buffer. The optimum MECK running buffer consisted of Borate 50 Mm; SDS 20 mM pH =9.6, unless stated otherwise. The wavelength for detection was set at 280 nm. The capillary had an effective length of 56 cm (total length: cm) and was operated at an applied voltage of 20 kV. 

Sample introduction was accomplished by vacuum injection for 30 s under a pressure of 50 psi (psi =6894.76 Pascals. Samples were ﬁltered through a 0.45-µm Whatman glass microﬁber ﬁlter before injecting into the CE instrument.

At the beginning of each day, the capillary was flushed for 10 min with 1N NaOH to activate the silanol groups of the capillary followed by 20 min of deionized water, and 5 min of running buffer. Before each sample injection, the capillary was rinsed for 5 min with 0.1N NaOH, followed by 5 min with the running buffer. In between runs, high pressure water was passed through the capillary for 10 min to flush out any impurities and to ensure a longer capillary life followed by 3 min of 1N NaOH, and 3 min of the running buffer to ensure reproducibility. At the end of the day, the capillary was flushed with 20 min of water followed by 20 min of air. Both ends of the capillary were dipped in deionized water before shutting down for the day.

## Results and Discussion

*Development of MEKC separation*

The chemical composition and concentration of running buffer could have signiﬁcant effects on MEKC separation .The selectivity in CE separation is greatly affected by pH of buffer solutions. In MEKC, the pH of the buffer solution affects the mobility, solubility and the partitioning of analytes into the micellar phase in the separation model. Typically, increasing buffer pH leads to the shortening of separation time (18).

In MEKC, separation can be achieved based on the differences in the distribution of the solutes between the hydrophobic and the charged micellar phase (41). Typically, the migration time for all the IFN increases with increasing SDS concentration, in a mixture of 10 mM phosphate and 10 mM borate buffer of pH 10.4. The migration order of the IFN changed slightly when the concentration of SDS was changed from 10 to 100 mM.

The unresolved pairs were later separated when the concentration of SDS was increased to50 mM and beyond. Therefore, the best resolution was obtained when SDS concentration was set at 50 mM in a buffer solution containing Borate 50 mM ; SDS 20 mM pH =9.6.

In addition to the running buffer conditions, the effect of separation voltage on the resolution was examined over the range of 10–30 kV in a buffer solution .Generally, an increase of EOF at high applied ﬁeld resulted in a shorter analysis time but also caused a decrease in the resolution for the separation of IFN. The reproducibility of the migration time of the IFN under optimum MEKC conditions was investigated by performing repeated injections (n = 3) of the IFN standards at a concentration of 250 ppm. The relative standard deviations (R.S.D.) for all they were in the range of 10.608%. The high reproducibility in migration time indicated that this method was reliable enough for the analysis of samples. Three independent injections were carried out for every calibration point. Based on the signal/noise ratio = 3, the limits of detection under optimum MEKC conditions were estimated to be in the range of Ca .6.5 µg/mL.

*Method validation*

**Figure 2:**
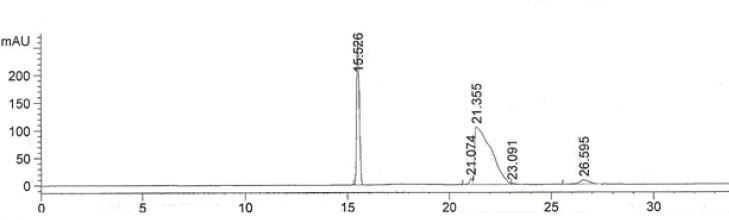
The electropherogram of 300 µg/mL IFN ß-1b solution

The developed method was validated by hydrodynamic injection for 30 seconds. The electropherogram of 300 µg/mL IFN ß1b solution is shown in Figure 2.

*Wavelength optimization in the separation*

**Figure 3:**
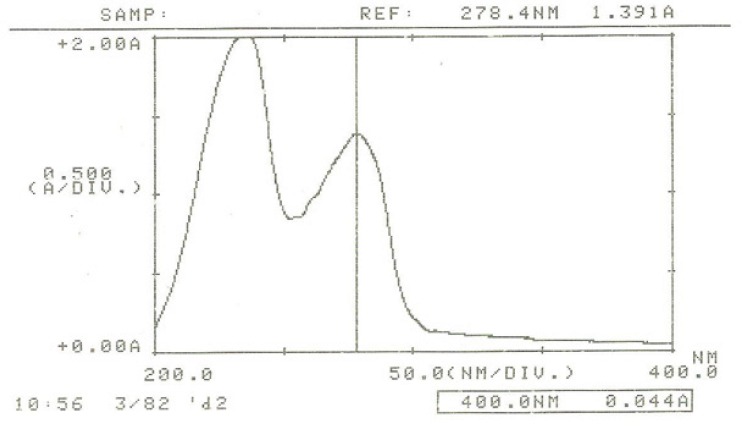
UV spectrum of IFN ß-1b

UV detector was set at 280 nm based on the ƛmax for IFN β-1b (Figure 3).

A calibration curve was constructed for the IFN ß-1b in buffer over the concentrations range of 164.70, 233.3, 480.2, 686 and 980 ppm.

**Figure 4:**
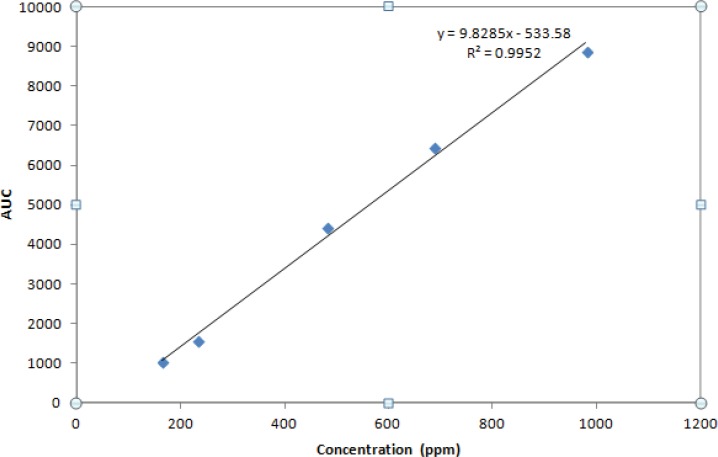
Calibration curve of IFN β-1b prepared by standard solutions

The within-day and day-to-day reproducibility expressed as relative standard deviation (RSD) were found to be less than 3.57 % and 3.68 % respectively. 

The accuracy of the method expressed as Relative Mean Error (RME) was ≤ 10.16 %. In our study, the results of precision and accuracy fulfilled the requirements. 

**Table 1:** Intra-day precision and accuracy of IFNβ-1b determination

**Added Concentration (ppm)**	**Added found concentration (ppm)(N=3)**	**Accuracy (%)**	**Precision (%) RSD**
100	114.71035 ± 1.3	114.7	1.2
300	287.6492335 ± 1.2	95.9	0.4
500	487.4267399 ± 15.4	97.4	3.2

**Table 2:** Inter-day precision and accuracy of IFN β-1b determination

**Added Concentration (ppm)**	**Added found concentration (ppm)**	**Accuracy (%)**	**Precision (%) RSD**
100	115.4±2.25	115.4	1.95
300	287.7±4.32	95.9	1.50
500	483.2±12.7	96.6	2.62

**Figure 5:**
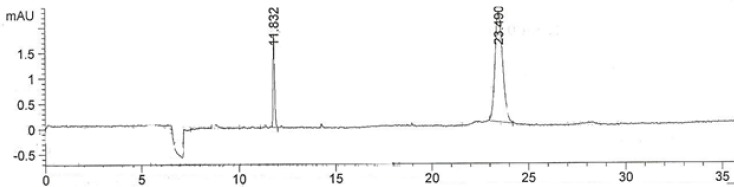
Electropherogram of LOQ=20 ppm

The effective LOQ of the assay, defined as the lowest quantifiable concentration with the variation of precision and accuracy ≤20% was found to be 20µg/mL which is equivalent to 12.3 ng IFN ß-1b (Figure 5). LOD was calculated as 1/3 of the LOQ (Ca .6.5 µg/mL).

**Figure 6:**
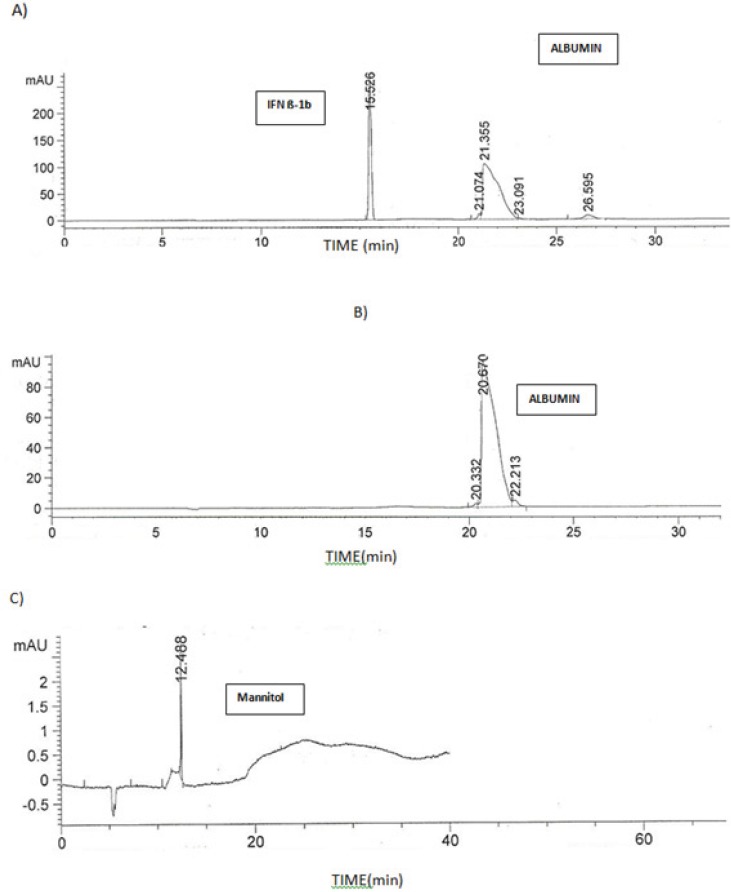
Electropherogram of A**)** solution of 300 µg/mL ZIFERON® B)solution of 12.5 mg/mL Albumin C)solution of 12.5 mg/mL Mannitol

The developed method was capabale of separating IFN β-1b from the other excipients in the formulation, *i.e*. albumin and Mannitol. Figure 6 compares the electropherogram obtained for ZIFERON® formulation with those obtained for albumin and Mannitol. 

*The effect of concentration on retention time*

**Figure 7:**
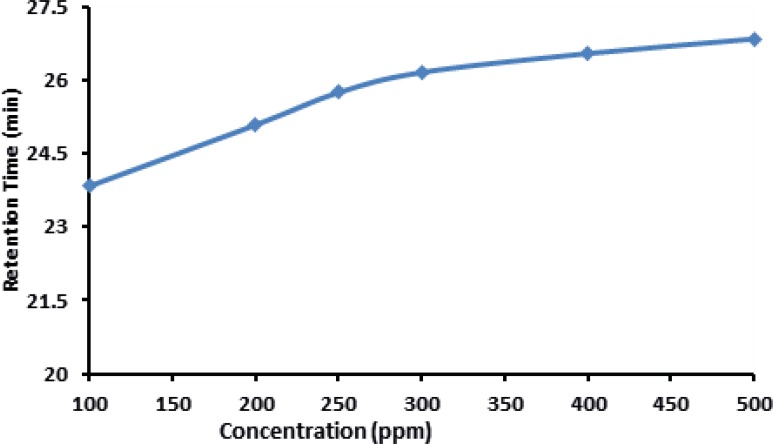
Effect of different concentration of IFNβ-1b on Retention Time in electropherogram

Retention time of IFN was slightly increased with increasing the concentration of IFN which is shown in Figure 7.

This could be the Influence of electro osmosis phenomenon on the mobility of the IFN in the buffer solution provided in this experiment. In case of the analysis of parenteral formulation of IFN ß-1b, we noticed that when high concentrations are used (>400 µg/mL ofIFN ß-1b) an abnormally high pressure is built up on the instrument which results in the blockade of the column. Therefore it is necessary to use low concentration for routine analysis to prevent any possible damage to the instrument. The same trend was observed when high temperatures (≥ 30c) were used .Therefore, column oven temperatures higher than 25 ˚C is not recommended for the analysis of IFN ß-1b formulations.

*The effect of pressure on retention time*

An experiment was performed to compare the effect of different injection pressures on the reproducibility and retention times for IFN ß-1b.

**Figure 8:**
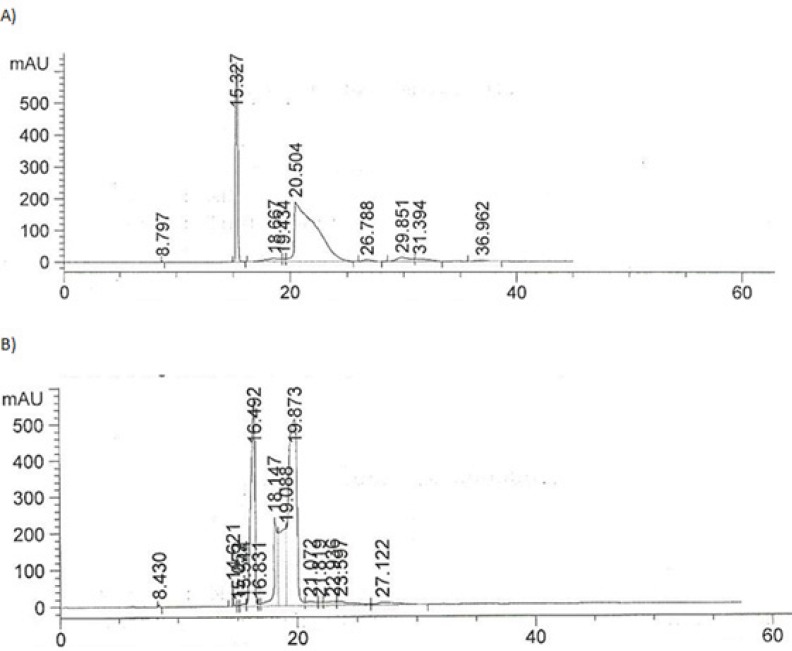
Comparison of electropherogram obtained at A) 50 mbar injection pressure and B) 100 mbar injection pressure.

The best injection pressures were found to be 50 and 75 mbar. Figure 8 compares the electropherograms obtained at 50 and 100 mbar.

The injection pressure of 50 mbar was chosen for the routine analysis of IFN ß-1b in parenteral formulation since it produced RSD values of less than 2% for the peak area of IFN ß-1b .

*The effect of repeated analyses on retention time*

In our hands, repeated analyses of the IFN ß-1b yielded basically the same profile but migration times successively increased as more runs were performed. These shifts in migration times were most probably caused by protein (mostly albumin) adsorption onto the capillary inner wall, which affects the magnitude of the EOF The higher concentration is used, the larger shift in retention time is observed.

*Matrix effect*

Sample matrix has practical implications on both resolution and quantization which may either improve or worsen the separation depending upon the CE conditions used. In MEKC, due to the low solubility of hydrophobic compounds most samples are prepared in fully organic solvent such as methanol or in a mixture of two solvent systems. For the sake of convenience, most samples are preferred to be diluted in aqueous rich media, but with aqueous media there is a lower resistance towards heat transfer compared to non-aqueous media and thus there will be higher Joule heating in using aqueous media. The preparation of samples in different media would give conductivity differences with respect to the separation buffer which would Influence the intensity of the peak area, peak height and separation efficiency (18-20).

Despite the advantages of using non–aqueous matrices in MEKC, we chose to use water as the solvent. Therefore the matrix of the samples will be identical with the matrix of parenteral formulation after reconstitution. 

*Analysis of real sample*

34 lyophilized parenteral preparations from 4 brands (one from Iranian company, Zistdaru-Danesh, brand name, ZIFERON® and the others imported from 3 foreign companies) were analyzed by validated method. The results were acceptable in potencies of formulations as 306.76 ± 30.09. 

## Conclusion

Capillary electrophoresis (CE) is a powerful separation tool, which in principle is very well suited for the analysis of proteins. Anew simple, rapid, precise and sensitive CE method has been established for assay of IFNβ-1 B in the bulk drug and in its parenteral formulation. Several properties of the method were monitored, including sensitivity, rapidity and repeatability of peak area, retention time and their RSD values. By use of a simple electrolyte IFNβ-1 B was successfully separated from albumin and other excipients in parenteral formulation.

Because the method has the advantage of rapidity, cost-effectiveness and simplicity, it is suitable for routine quality control analysis of IFN ß-1b 1 formulation and could be a proper alternative for HPLC and LC-MASS method.
